# The role of radial head morphology in proximal radioulnar joint congruency during forearm rotation

**DOI:** 10.1002/jeo2.70059

**Published:** 2024-10-21

**Authors:** Hailong Zhang, Guang Yang, Yi Lu

**Affiliations:** ^1^ Department of Sports Medicine Capital Medical University Affiliated Beijing Jishuitan Hospital Beijing China

**Keywords:** congruency, morphology, pronation, proximal radioulnar joint, supination

## Abstract

**Purpose:**

Congruency of the proximal radioulnar joint (PRUJ) is important in the rotation of the forearm while any compromise would significantly impair the elbow function. The purpose of this study is to determine the morphological features of the radial head and investigate its role in the congruency of the PRUJ during forearm rotation. The hypothesis is that the PRUJ is more congruent in the maximal pronation and supination positions than in the neutral position.

**Methods:**

Thirty sets of computed tomography images of the elbow were acquired, and a three‐dimensional model of the proximal radius and ulna was generated. The radius of curvature of the radial head and the radial head at the maximal pronation, neutral positions and maximal supination were calculated and compared with a one‐way analysis of variance.

**Results:**

The point on the radial head contacting the middle point of radial head at the maximal pronation and supination was located at the ends of the semi‐major axis of the ellipse, while it was located at one end of the semi‐minor axis at neutral position. The radii of curvature of the pronation, neutral and supination points of the radial head were 14.72 ± 1.51, 9.74 ± 1.49 and 14.58 ± 1.70 mm, respectively. The value of the neutral point was significantly smaller than that of the pronation and supination points.

**Conclusions:**

This study quantitatively evaluated the morphology of the radial head and suggested that the best congruency of the PRUJ was achieved at maximal pronation and supination, while the neutral position was associated with the least congruency.

**Level of Evidence:**

Basic Science Study.

## Graphical abstract

This study quantitatively evaluated the morphology of the radial head and suggested that the best congruency of the proximal radioulnar joint (PRUJ) was achieved at maximal pronation and supination, while the neutral position was associated with the least congruency. Our study may improve the understanding of the biomechanics of the PRUJ during forearm rotation and aid in anatomical radial head reconstruction and prosthesis design.

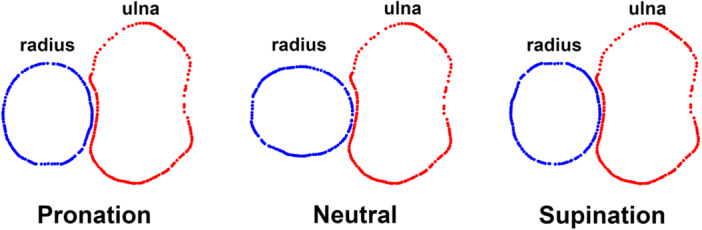

**Abbreviations**
3D: three‐dimensional
CT: computed tomography
IRB: institutional review board
NP: neutral point
PP: pronation point
PRUJ: proximal radioulnar joint
RN: radial notch of the ulna
SD: standard deviation
SP: supination point

## INTRODUCTION

The elbow is a complex joint composed of the radiohumeral, ulnohumeral and proximal radioulnar joints (PRUJ). The PRUJ, together with the interosseous membrane and distal radioulnar joint, contributes to the pronation and supination of the forearm [16]. The radial head plays a paramount role in the function of PRUJ as it can freely rotate around the axis of forearm rotation to facilitate pronation and supination [22]. Fracture or pathology of the radial head significantly alters its morphology and impairs the congruency of the PRUJ [3, 5, 23]. Even though much attention has been paid to the congruency of the PRUJ, few results have been published on the exact morphological nature of the peripheral part of the radial head, which plays an important role in forearm rotation, as well as design of prosthesis of radial head [4, 14].

In the past, the cross‐section of the radial head was regarded as circular, which was supposed to generate a uniform contact area between the radial head and RN regardless of the rotational position of the forearm [15]. Recently, an increasing number of results have suggested that the radial head is elliptical rather than perfectly circular and that the PRUJ congruency changes during forearm rotation [4, 24]. Some studies have also implied that this morphological pattern may be associated with the unique biomechanics of the PRUJ [14]. When radial head replacement was carried out using an anisotropic anatomical radial head prosthesis, proper orientation during the prosthesis placement would be of paramount importance to restore this congruency. Thus, a clear understanding of the relationship between the morphology of the radial head and the congruency of the PRUJ during forearm rotation is needed.

This study aimed to determine the morphological features of the radial head and investigate its role in the congruency of the PRUJ during forearm rotation. The hypothesis is that the PRUJ is more congruent in the maximal pronation and supination positions than in the neutral position.

## METHODS

### Image acquisition and 3D CT model generation

**Figure 1:**
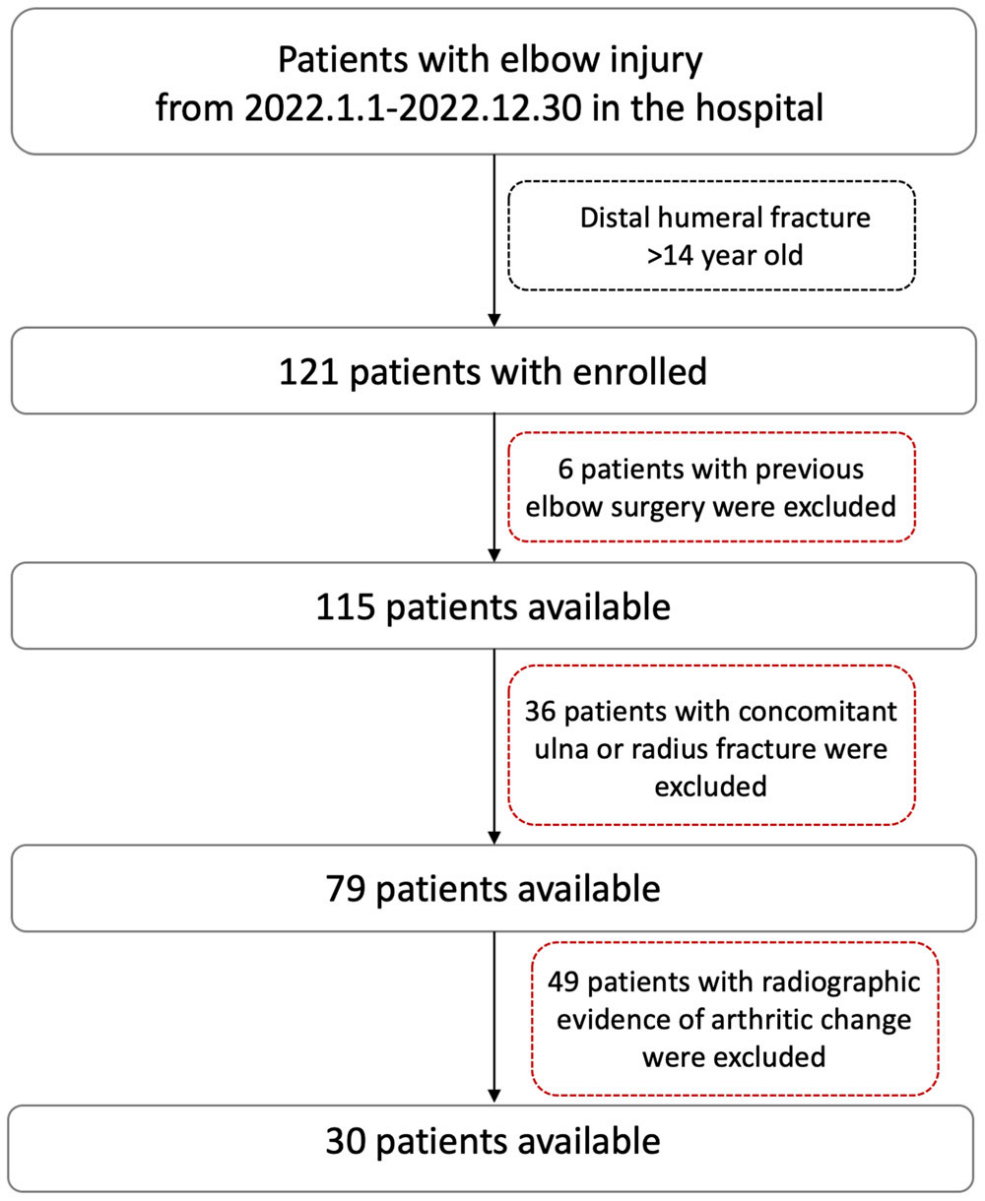
Flow chart of subject inclusion.

Following approval by our institutional review board (IRB) (No. 202115239), a total of 30 patients with distal humerus fracture (13 females and 17 males) aged 25–41 years (mean, 32.7 years) were enroled in the study. The exclusion criteria included a history of previous elbow surgery, concomitant fracture of proximal ulna and radius or radiological evidence of arthritic change involving elbow joint (Figure 1). Body mass index was not taken into consideration in this study. Informed consent was obtained from each subject according to the IRB guidelines.

**Figure 2:**
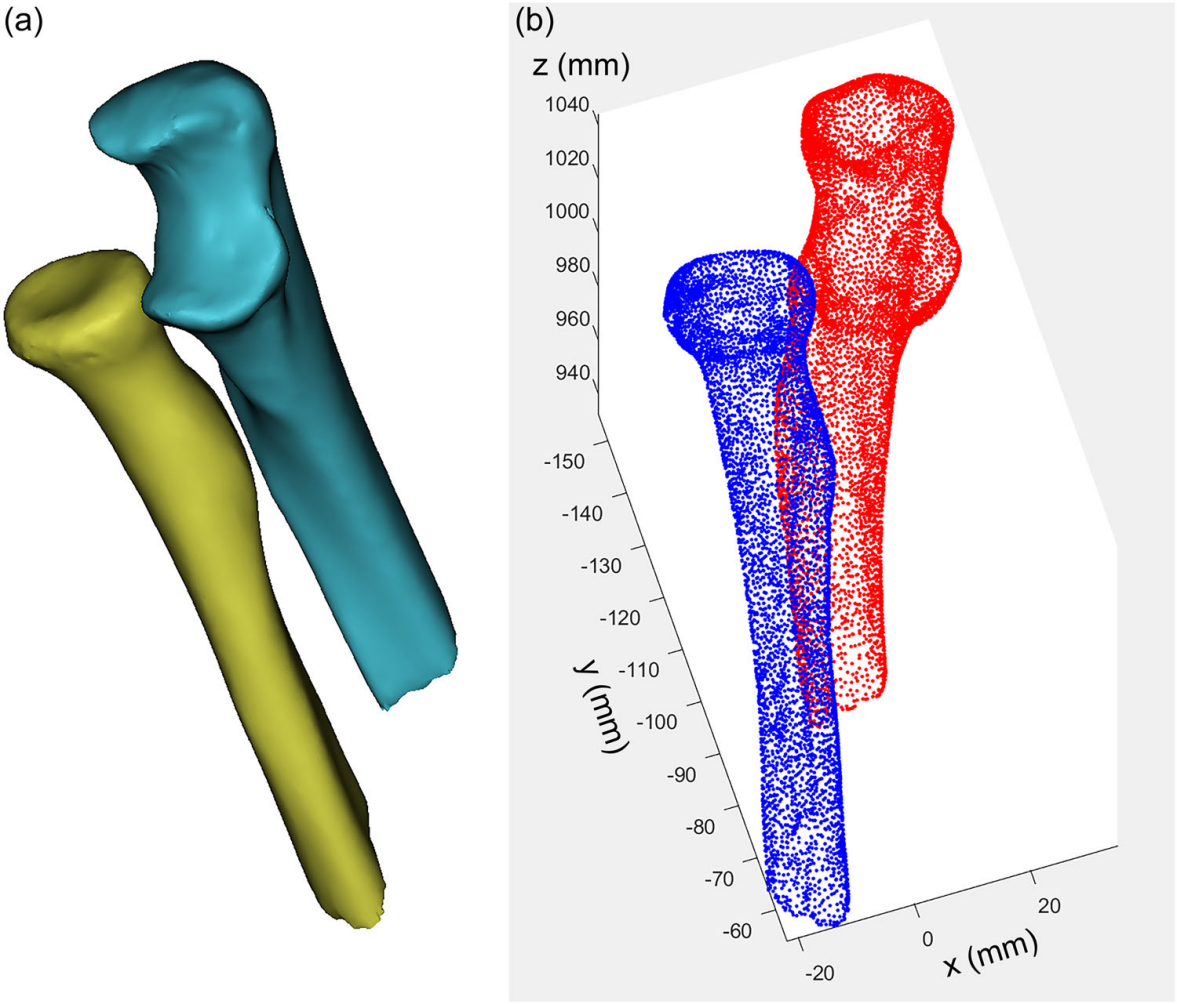
Three‐dimensional (3D) geometry of the proximal radius and ulna. (a) 3D contour of the proximal radius and ulna were reconstructed from computed tomography images. (b) A point‐cloud model was generated.

Data were collected using a helical computed tomography (CT) scanner (Aquillion 64, Toshiba America Medical Systems). CT scans of the elbows were performed at the neutral position with a slice thickness of 0.8 mm and a resolution of 512 × 512 pixels using the standard 120 kVp and 200 mA bone‐reconstruction sequence. The proximal radius and ulna were extracted from the CT images, and the three‐dimensional (3D) geometry was reconstructed using commercial 3D reconstruction software (Mimics, Materialise Inc.), which was then exported in a point‐cloud format (Figure 2).

### Establishment of the local coordinate system

**Figure 3:**
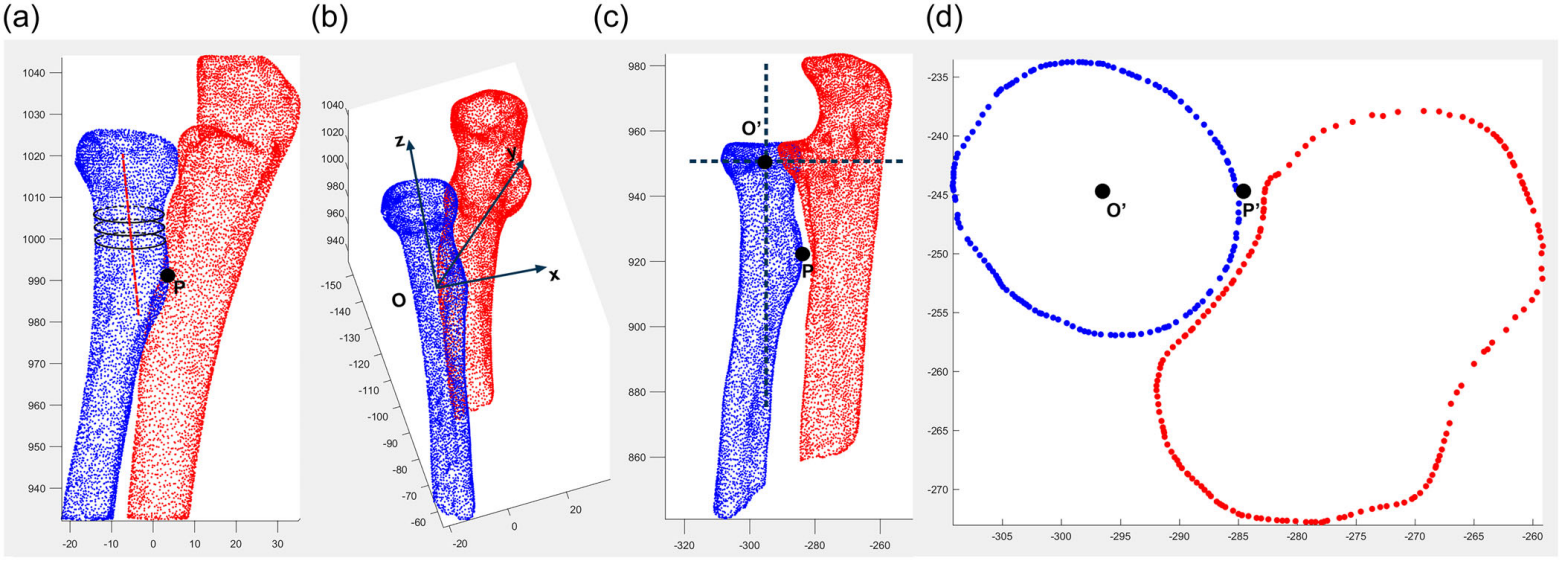
Reorientation of the proximal radius and ulna and generation of boundary points on a standard cross‐section. (a) The most prominence of radial tuberosity was identified and labelled as point P. The central axis of the radius was determined by fitting the coordinates of the centres in three sequential cross‐sections (black circles) within this segment, which was defined as the *Z*‐axis (red line). (b) The intersection between the *Z*‐axis and a plane passing through point P and perpendicular to the *Z*‐axis was defined as the coordinate centre O. A line perpendicular to the plane passing through point P and the *Z*‐axis was defined as the *Y*‐axis. The *X*‐axis was defined as the line perpendicular to both the *Y*‐ and *Z*‐axes. (c) The point‐cloud model was rotated with a coordinate‐transformation algorithm and was projected onto the *XZ* plane to show the point O′, which was defined as the intersection of the *Z*‐axis at the level of the middle of the radial head. (d) The points on the cross‐section at the level of the middle of the radial head were extracted and projected onto the *XY* plane, with point O′ labelled as mentioned in panel (c). The boundary of the radius (blue dots) and ulna (red dots) were illustrated and the projection of point P on this cross‐section was labelled as point P′.

To investigate the role of radial head morphology in PRUJ congruency during forearm rotation, it was necessary to determine the kinematic axis of forearm rotation. As indicated by Oki et al., the forearm rotational axis was closest to the principal axis of inertia at 16% of the length of the proximal radius, which could be anatomically marked by the distal end of the biceps brachii attachment [19]. The prominence of the radial tuberosity was identified in the 3D model, and the segment between the radial head and the level of the most prominence of radial tuberosity was selected (Figure 3a). The central axis of forearm rotation, approximated by the principal axis of inertia at 16% of the length of the proximal radius, was determined using the approach described by Zhang et al., that is, by fitting the coordinates of centres in three sequential cross‐sections within this part [25] (Figure 3a).

A local coordinate system was established for the subsequent evaluation of the morphology of the radial head (Figure 3b). The established central axis of the proximal radius was defined as the *Z*‐axis. Origin O was set as the intersection point of the line passing through point P and the line perpendicular to the *Z*‐axis. The *Y*‐axis was defined as the vector perpendicular to the plane passing through the *Z*‐axis and point P. A vector perpendicular to both the *Y*‐ and *Z*‐axes was set as the *X*‐axis. A coordinate transformation algorithm was developed by reorientation of the proximal ulna and radius with respect to local coordinates. Points from the radius and ulna at the middle level of the radial head were selected, which were projected onto the standard cross‐section, that is, the *XY* plane, for morphological analysis, and the projections of points O and P were labelled as points O′ and P′ (Figure 3c,d).

### Calculation of the radius of curvature and the arc of the RN

**Figure 4:**
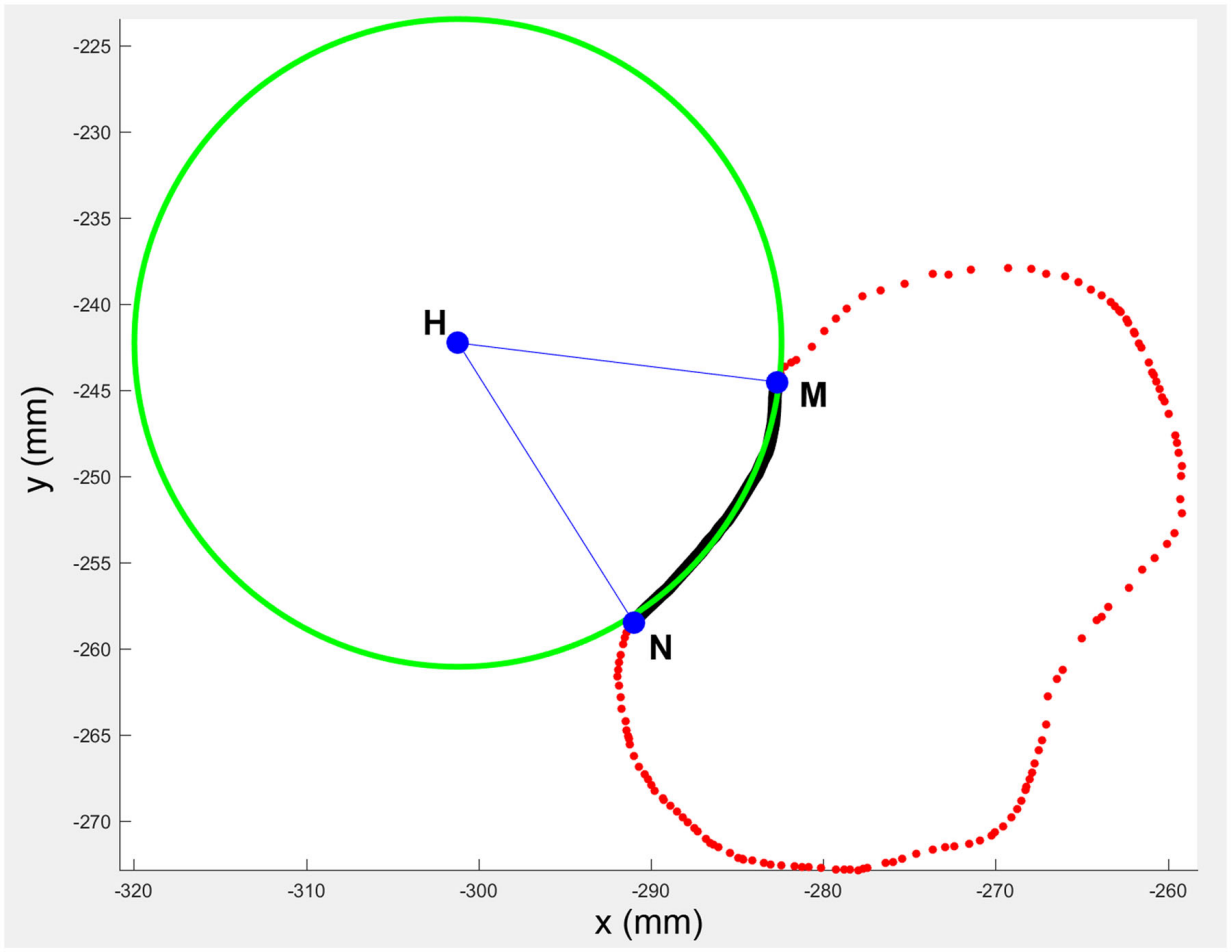
Fitting of the boundary points of RN with a best‐fit circle. The boundary points on RN were marked (black curve), with the volar and dorsal ends labelled as points N and M. A best‐fit circle of these points (green circle) was generated with its centre marked as point H. RN, the radial notch of the ulna.

Following the projection of the points at the middle level of the radial head on the XY plane, the projected points at the RN were selected and a best‐fit circle was generated based on the distribution of the points. The radius of curvature of RN was calculated from the radius of the best‐fit circle, and the arc of RN was subtended by the arc at the centre of the best‐fit circle and expressed as ∠MHN (Figure 4).

### Calculation of the radius of curvature on the border of radial head

**Figure 5:**
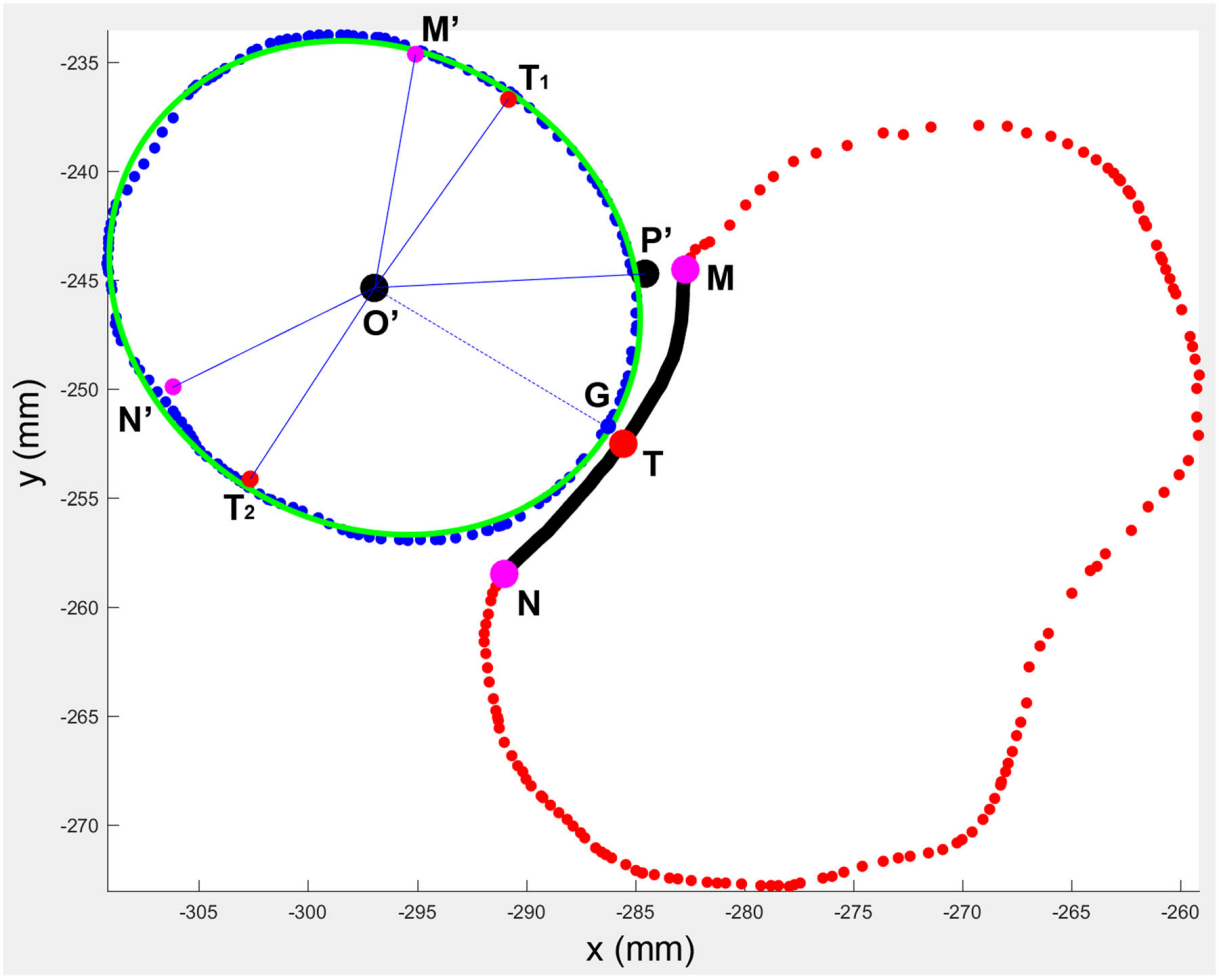
Best‐fit ellipse fitting of the radial head and determination of points in contract with the RN during forearm rotation. The boundary of points demarcating the radial head and ulna were illustrated (blue dots and red dots). The boundary of RN was marked (black curve) with its volar end, dorsal end and middle point labelled separately (points N, M and T, respectively). The points O′ and P′ were the projections of the *Z*‐axis and the most prominence of the radial tuberosity, and the line O′P′ indicated the *X*‐axis. The end of the safe zone at maximal supination was marked as point M′. T1 was the point in contact with T at maximal supination (SP). Points N′ and T2 (PP) were in contact with points N and T at maximal pronation, respectively. The point on the radial head in contact with point T on RN at a neutral position was defined as point G (NP). NP, the point on the radial head in contact with the middle point of the radial notch of the ulna at neutral position; PP, the point on the radial head in contact with the middle point of the radial notch of the ulna at maximal pronation; RN, the radial notch of the ulna; SP, the point on the radial head in contact with the middle point of the radial notch of the ulna at maximal supination.

Points on the border of the radial head on the projection were selected, and a best‐fit ellipse was generated accordingly. The semi‐major and semi‐minor axes were derived and marked as *a* and *b*, respectively. The point of the radial head in contact with the volar rim of the PRUJ at the maximal pronation was labelled as point M′, and the angle ∠M′O′P′ indicating its location with the reference to the most prominence of radial tuberosity was suggested to be 84° [7] (Figure 5). The point on the radial head in contact with the middle point of RN at the maximal pronation was named the pronation point (PP) and labelled as point T_1_, and the angle ∠T_1_O′P′ indicating its location with the reference to the most prominence of radial tuberosity was expressed as

$$∠T_{1}O\prime P\prime =∠M\prime O\prime P\prime -\frac{1}{2}\times ∠\mathrm{MHN}.$$

The point of the radial head in contact with the dorsal rim of the PRUJ at maximal supination was labelled as point N′, and the angle ∠N′O′P′ indicating its location with the reference to the most prominence of radial tuberosity was suggested to be 154° [7] (Figure 5). The point on the radial head in contact with the middle point of RN at maximal supination was named the supination point (SP) and labelled as point T_2_, and the angle ∠T_2_O′P± indicating its location with the reference to the most prominence of radial tuberosity was expressed as (Figure 5)

$$∠T_{2}O\prime P\prime =∠N\prime O\prime P\prime -\frac{1}{2}\times ∠\mathrm{MHN}.$$

The point on the radial head in contact with the middle point of RN at the neutral position was named the neutral point (NP) and labelled as point G. The range of supination was approximately 6° larger than that of pronation, at 90° elbow flexion [20, 26]. The angle ∠GO′P′ indicating its location with reference to the most prominence of radial tuberosity was expressed as (Figure 5)

$$∠\mathrm{GO}\prime P\prime =∠T_{2}O\prime P\prime -(\frac{1}{2}\times ∠\mathrm{MHN}-3).$$

The radius of curvature of any point on the ellipse was derived from the equation

$$R(\theta )=\frac{{\left(\sqrt{{\left(b\cos\theta \right)}^{2}+{(a\sin\theta )}^{2}}\right)}^{3}}{\mathrm{ab}},$$

where *a* and *b* are the semi‐major and semi‐minor axes, respectively, and θ is the angle defined by the location of the point in the reference of the semi‐major axis.

### Statistical analysis

Nonlinear fitting was used to generate the best‐fit circles and ellipses. All values were expressed as mean ± standard deviation (SD) and normality of the data was confirmed using Shapiro‐Wilk test. For comparison of the maximal and minimal diameters and radii of curvature of the radial head, as well as the localization of PP, NP or SP with the points of the semi‐major axis or semi‐minor axis, a one‐sample Student's *t* test was used to quantify significant differences. For comparison of the radii of curvature of PP, NP, SP and the curvature of RN, repeated measures one‐way analysis of variance was used, and the Tukey post hoc honestly significant difference test was used to compare values amongst groups, if present. Statistical significance was set at p < 0.05. To compare the maximal radius of curvature of the best‐fit ellipse and RN, a prior experiment was performed for preliminary estimates of the mean and SD, and a sample size of 30 patients was adequate to achieve 80% power for the comparison.

## RESULTS

### Morphological description of RN

RN was fitted by a best‐fit circle (*p* < 0.001). The radius of curvature was 17.48 ± 2.33 mm and the radial notch angle was 58.32 ± 6.74°.

### Morphological description of the radial head

The radial head was fitted using a best‐fit ellipse (*p* < 0.001). The semi‐major and semi‐minor axes of the ellipse were 23.84 ± 1.73 and 20.69 ± 1.43 mm, respectively, with significant differences (*p* < 0.001). The points with the maximal and minimal radii of curvature of the radial head were at the ends of semi‐major and semi‐minor axis, with the values of 14.72 ± 1.93 and 9.73 ± 1.22 mm, respectively. A significant difference was observed between the maximal and minimal radii of curvature of the radial head (*p* < 0.001). The positions of the two ends of the semi‐major axis with reference to the most prominence of radial tuberosity were −131.26 ± 5.44° and 50.32 ± 6.19°, respectively. The positions of the two ends of the semi‐minor axis with reference to the most prominence of radial tuberosity were −41.92 ± 4.37° and 140.54 ± 5.72°, respectively.

### Localization of the contact point of the radial head at different positions of rotation

**Figure 6:**
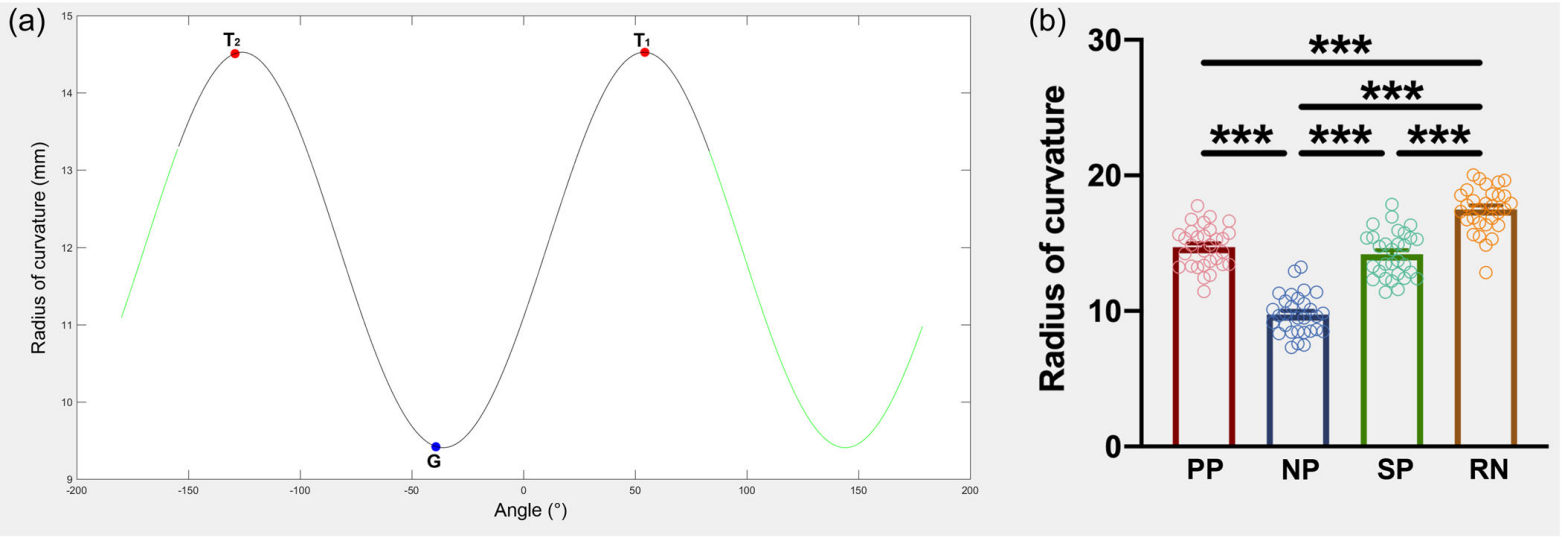
The radius of curvature of point on radial head contacting the middle point of RN during forearm rotation. (a) The distribution of the radius of curvature on the boundary of the radial head was shown from −180° to 180°. The horizontal axis indicated the location of the points on the boundary of the radial head. The vertical axis indicated the radius of curvature. The two segments of the curve in green indicated the safe zone, while the segment marked in black indicated the portion of the radial head in contact with RN during forearm rotation. T2, T1 and G showed the radii of curvature of PP, NP and SP, respectively. (b) The radii of curvature of PP, NP and SP were significantly smaller than the radius of curvature of RN. The radius of curvature of NP was significantly smaller than that of PP and SP. NP, the point on the radial head in contact with the middle point of the radial notch of the ulna at neutral position; PP, the point on the radial head in contact with the middle point of the radial notch of the ulna at maximal pronation; RN, the radial notch of the ulna; SP, the point on the radial head in contact with the middle point of the radial notch of the ulna at maximal supination. ***, *p* < 0.001.

**Figure 7:**
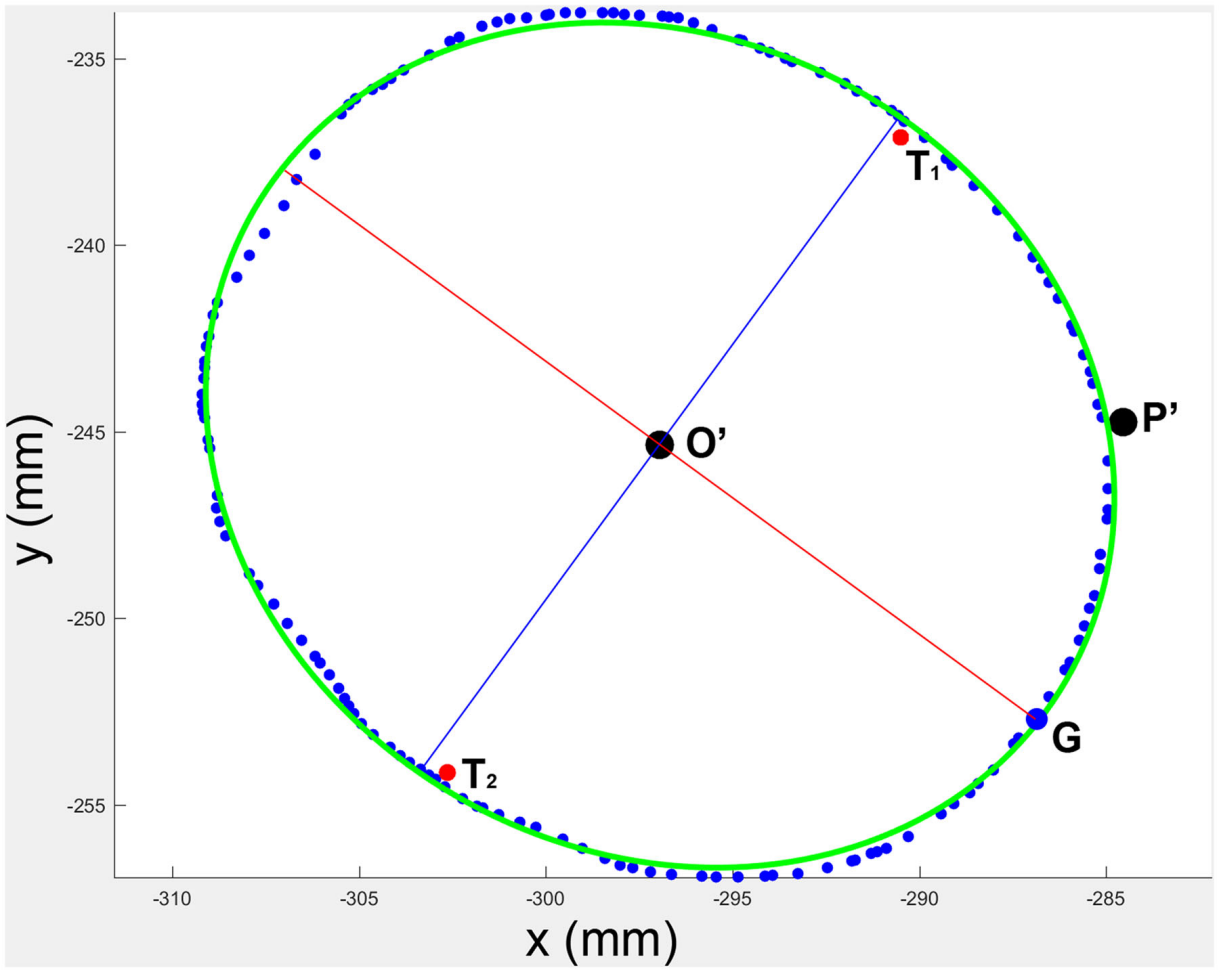
Relationship between the location of PP, NP and SP and the ends of semi‐major or semi‐minor axis. The boundary of points demarcating the radial head and ulna were illustrated (blue dots and red dots). The points O′ and P′ were the projections of the *Z*‐axis and the most prominence of radial tuberosity. T1, G and T2 were the points in contact with the middle point on RN at maximal supination (SP), neutral position and maximal pronation. The semi‐major axis and the semi‐minor axis were marked in red and blue lines. Overlapping of PP and SP with the ends of the semi‐major axis and NP with the end of the semi‐minor axis were shown, respectively. NP, the point on the radial head in contact with the middle point of the radial notch of the ulna at neutral position; PP, the point on the radial head in contact with the middle point of the radial notch of the ulna at maximal pronation; RN, the radial notch of the ulna; SP, the point on the radial head in contact with the middle point of the radial notch of the ulna at maximal supination.

The location of PP indicated by the angle with reference to the most prominence of radial tuberosity was −124.84 ± 3.37° (Figure 6a). The locations of NP and SP were described in the same manner and were −38 ± 3.37° and 54.84 ± 3.37°, respectively (Figure 6a). No significant differences were found between PP and the end of the semi‐major axis (*p* = 0.46), NP and the end of the semi‐minor axis (p = 0.33), as well as SP and the end of the semi‐major axis (p = 0.19) (Figure 7).

### Calculation of the radii of the curvature of the radial head at different positions of rotation

The radius of the curvature on the radial head contacting the RN decreases when the forearm rotates from the maximal pronation to neutral position, and then increases from neutral position to the maximal supination (Table 1, Figure 6a). The radii of curvature of PP, NP and SP were 14.72 ± 1.51, 9.74 ± 1.49, and 14.58 ± 1.70 mm, respectively. The radii of curvature of PP and SP were significantly smaller than the radius of curvature of RN (df = 3, *F* (2.846, 82.53) = 111.7, p < 0.001; for comparison between PP and radial notch, *p* < 0.001; between NP and radial notch, *p* < 0.001; and between SP and radial notch, *p* < 0.001) (Figure 6b). Concerning the radius of curvature of the radial head, the value of NP was significantly smaller than that of PP and SP (for comparison between PP and NP, p < 0.001; and between SP and NP, *p* < 0.001) (Figure 6b). No significant difference was found in the radius of curvature of PP and SP (*p* = 0.23).

**Table 1:** Radius of curvature of points on the radial head contacting the middle point of radial notch during forearm rotation.

Radius of curvature (mm)	Pronation	Supination
Neutral	9.74 ± 1.49	9.74 ± 1.49
10°	9.87 ± 1.34	9.85 ± 1.41
20°	10.25 ± 1.46	10.22 ± 1.43
30°	10.91 ± 1.28	10.86 ± 1.38
40°	11.84 ± 1.44	11.81 ± 1.32
50°	12.64 ± 1.33	12.62 ± 1.26
60°	13.51 ± 1.26	13.49 ± 1.24
70°	14.08 ± 1.35	14.03 ± 1.36
80°	14.47 ± 1.39	14.38 ± 1.28
Maximal	14.72 ± 1.51	14.58 ± 1.70

## DISCUSSION

In this study, the morphology of the radial head was quantitatively evaluated using a 3D model of the proximal radius and ulna. The radius of curvature of RN and boundary of the radial head at different rotational states were matched and analysed. As hypothesized, the PRUJ demonstrated better congruency in the maximal pronation and supination positions than in the neutral position.

After creation and re‐orientation of the 3D model following the coordinate transformation algorithm, the cross‐section at the middle level of the radial head was selected, and the morphology of RN was analysed. In a previous study, RN was fitted as a circle with a PRUJ coverage between 69° and 79°, but information on other parameters was still lacking [14]. Using the 3D model, RN could be fitted with a best‐fit circle with a radius of curvature of 17.15 ± 2.33 mm and a coverage of 58.32 ± 6.74°. The cross‐section selected in our study was different from that in the previous study, which may explain the relatively smaller value of the coverage.

Precise and quantitative characterization of the shape of radial head is paramount in reconstruction of radial head with prosthesis. Anatomically restored the radial head could mimic the elbow kinematics, joint pressure, and interosseous membrane tension in physiological conditions [12, 21]. An appropriate selection of radial head prosthesis results in joint instability or abnormal pressure within the ulnoradial joint, which would be associated with capitellar degeneration or implant failure [2, 8, 18].

Several studies have been conducted to quantitatively describe the morphology of the radial head to restore or reconstruct the original anatomy in cases of radial head pathology [4, 9]. Most recent reports have suggested that the shape of the radial head is elliptical rather than perfectly circular [4, 9]. Bachman's study found elliptical design of radial head better radiocapitellar contact area and decreased contact pressures during compressive loading [1]. In our study, the radial head could be successfully fitted by a best‐fit ellipse, the semi‐major and semi‐minor axes of which were 23.84 ± 1.73 and 20.69 ± 1.43 mm, respectively, which was consistent with previous results.

Although it is accepted that the radial head has an oval shape, few studies have been conducted to investigate the direction of the long axis of the ellipse and the reason for this configuration. Previous investigations mainly focused on the area on the periphery of the radial head called the ‘safe zone’, which is important for implant placement, but little attention has been drawn to the part in direct contact with the RN [13, 17]. Deschrijver et al. suggested that in neutral position, the angle between the long axis of radial head and the forearm plane is 5.28° and between the long axis of radial head and the PRUJ is 33.46° [9]. However, detailed information concerning the morphology of the radial head and its role in the congruency of the PRUJ was lacking. The current study suggested that the point on the radial head in contact with RN at maximal pronation and supination approximated the ends on the semi‐major axis and demonstrated a larger radius of curvature, which showed better congruency of the PRUJ. The point on the radial head in contact with RN in the neutral position approximated the ends on the semi‐minor axis and showed a smaller radius of curvature and less congruency of the PRUJ. The direction of the semi‐major axis in our study is similar to that of the long axis of radial head in the previous result [9]. Furthermore, our study also indicated that at maximal pronation and supination, the increased congruency of the PRUJ may produce better stability, while in the neutral position, the mismatch of the radius of curvature between the radial head and radial tuberosity of the ulna would allow for motion of the radial head confined by RN and the annular ligament. This may explain the findings of Galik et al. that the anterior/posterior and medial/lateral movement of the radial head occurred in the axial plane at the PRUJ during forearm rotation in cadavers [10].

As suggested in this study, PP and SP approximate the ends of the semi‐major axis and NP approximates the end of the semi‐minor axis. Thus, the orientation of the ellipse generated from the contour of the radial head may also be associated with forearm rotation, which has rarely been mentioned in the literature [14]. Reconstruction of the radial head using biomaterial or prosthesis in the shape of ellipse was popularized in clinical practice, but if it was placed without consideration of the anatomical orientation, instability of PRUJ and subluxation would be a potential concern after the surgery and may need revision [6]. In this study, orientation of the ellipse was determined by localizing the ends of the semi‐major and semi‐minor axes in reference to the radial tuberosity, which provided feasible way for accurate placement of the reconstructed radial head during the operation.

The results of this study quantitatively describe the morphology of the radial head, which has several clinical relevance. First, an anatomical radial head prosthesis would better restore the original bone architecture, as the result of this study suggested the shape of the radial head is elliptical rather than circular. Second, the orientation of the anatomical radial head prosthesis in operation should be carefully planned, as inappropriate placement of the prosthesis would impair the rotation of the forearm due to a mismatch of the curvature of the sigmoid notch and various locations of the radial head.

This study had several limitations. First, the average value of the safe zone was used to determine the point of the radial head in contact with the RN during the forearm rotation. However, this parameter varies among individuals. The determination of a case‐specific safe zone may improve the identification of matching points between the radial head and RN during forearm rotation. Second, the study was based on the bone architecture of the proximal radius and ulna from CT scans, and cartilage was not considered [11]. Further studies are required to analyse the role of the cartilage in the congruency of the PRUJ. Third, the role of the ligament and tendon during the forearm rotation was not taken into consideration. However, several previous studies focused on the bony architecture of radial head morphology have already suggested this elliptical characteristic.

## CONCLUSION

This study quantitatively evaluated the morphology of the radial head and suggested that the best congruency of the PRUJ was achieved at maximal pronation and supination, while the neutral position was associated with the least congruency.

## AUTHOR CONTRIBUTIONS

All authors contributed to the study's conception and design. Hailong Zhang, Guang Yang and Yi Lu performed material preparation, data collection and analysis. Hailong Zhang wrote the first draft of the manuscript, and all authors commented on previous versions. All authors read and approved the final manuscript.

## CONFLICT OF INTEREST STATEMENT

The authors declare no conflict of interest.

## ETHICS STATEMENT

Ethical approval was obtained from the institutional review board (IRB) of Beijing Jishuitan Hospital (No. 202115239). Informed consent was obtained from all individual participants included in the study. Patients signed informed consent regarding publishing their data and photographs.

## Data Availability

The data that support the findings of this study are available on request from the corresponding author Yi Lu.
